# Mathematical models of amino acid panel for assisting diagnosis of children acute leukemia

**DOI:** 10.1186/s12967-019-1783-9

**Published:** 2019-01-23

**Authors:** Zhidai Liu, Tingting Zhou, Xing Han, Tingyuan Lang, Shan Liu, Penghui Zhang, Haiyan Liu, Kexing Wan, Jie Yu, Liang Zhang, Liyan Chen, Roger W. Beuerman, Bin Peng, Lei Zhou, Lin Zou

**Affiliations:** 1 Clinical Center for Molecular Medicine, Children’s Hospital of Chongqing Medical University, 136 Zhongshan 2 Rd, Chongqing, 400014 China; 2 Department of Endocrinology and Metabolism, The Second Affiliated Hospital of Chongqing Medical University, Chongqing, China; 3 Key Laboratory of Clinical Laboratory Diagnostics (Ministry of Education), College of Laboratory Medicine, Chongqing Medical University, Chongqing, China; 4 Clinical Laboratory Center, Children’s Hospital of Chongqing Medical University, Chongqing, China; 5 Chinese Ministry of Science and Technology Demonstration Base for International Cooperation, Beijing, China; 6 The Development and Diseases Key Laboratory of Ministry of Education, Nanning, China; 7 The Pediatrics Key Laboratory of Chongqing Science and Technology Committee, Chongqing, China; 8 Department of Statistics and Applied Probability, Faculty of Science, National University of Singapore, Singapore, Singapore; 9 Singapore Eye Research Institute, The Academia, 20 College Road, Singapore, 169856 Singapore; 10 Department of Ophthalmology, Yong Loo Lin School of Medicine, National University of Singapore, Singapore, Singapore; 11 Ophthalmology and Visual Sciences Academic Clinical Research Program, Duke-NUS Graduate Medical School, Singapore, Singapore; 12 Department of Hematology, Children’s Hospital of Chongqing Medical University, Chongqing, China; 13 Department of Health Statistics, School of Public Health, Chongqing Medical University, Yuzhong District, Chongqing, China

**Keywords:** Acute leukemia, Mathematical model, Amino acid panel, Mass spectrometry

## Abstract

**Background:**

The altered concentrations of amino acids were found in the bone marrow or blood of leukemia patients. Metabolomics technology combining mathematical model of biomarkers could be used for assisting the diagnosis of pediatric acute leukemia (AL).

**Methods:**

The concentrations of 17 amino acids was measured by targeted liquid chromatograph–tandem mass spectrometry in periphery blood collected using dried blood spots. After evaluation, the mathematical models were further evaluated by prospective clinical validation cohort for AL diagnosis.

**Results:**

The concentrations of 13 in 17 amino acids were statistically different between the periphery blood dried serum dots measured by targeted LC–MS/MS. The receiver operating characteristic analysis for the models of amino acid panel showed that the area under curve for AL diagnosis were 0.848, 0.834 and 0.856 by SVM, RF and XGBoost. The *Kappa* values in further prospectively evaluated clinical cohort were 0.697, 0.703 and 0.789 (*p *> 0.05) respectively, and the accuracies for the models were 84.86%, 85.20% and 89.46% respectively with further clinical validation.

**Conclusions:**

The established mathematical model is a faster, cheaper and more convenient way than conventional methods, and no significant difference on the effect of diagnosis comparing with conventional methods. The mathematical model can be clinically useful for assisting pediatric AL diagnosis.

**Electronic supplementary material:**

The online version of this article (10.1186/s12967-019-1783-9) contains supplementary material, which is available to authorized users.

## Background

Acute leukemia (AL) is the most common cancer in children under 15 years of age, divided into acute lymphoblastic leukemia (ALL) and acute myeloid leukemia (AML), which ALL accounts for 60–70% and AML for 30–40% [1]. The diagnosis of AL is dependent on the multiple laboratory tests, which require the combination of assays of morphological, immunological, cytogenetic and molecular (MICM) inputs [2]. The current procedure (MICM assays) of using bone marrow cells from AL patients is painful and inconvenient for children [3]. The immunological tests rely on flow cytometry, while the molecular tests, such as, reverse transcription polymerase reaction (RT-PCR) and high throughput sequencing are used to measure fusion genes and key mutations of the driven genes. All the tests are instrument-dependent and the proper interpretation of results is required. There are increasing interests in discovering the new sensitive and specific biomarkers in the peripheral blood (PB) as an easy way to assist AL diagnosis.

The connection between nutrient metabolites and cancers has been reported extensively [4]. The metabolic environment is essential for cancer cell growth [5] and the metabolomics analysis of samples from cancer patients, including leukemia, enables the identification of novel specific biomarkers [6]. Although most scientists focused on determining the relationship between glucose metabolism and different cancers [7], the occurrence and development of leukemia has been shown to be closely related to amino acid metabolism that affects the protein synthesis. For example, proline disturbs several key metabolic pathways to promote the disease progress and affects the treatment of leukemia [8]. Besides, others’ report have proven that the amino acids were related with cell proliferation, apoptosis or drug treatment of different cancers [9–15]. Therefore, in this study, we aimed to determine whether the alterations of amino acid concentrations could be useful for the diagnosis of AL.

For measuring multiple amino acids simultaneously, the targeted liquid chromatograph–tandem mass spectrometry (LC–MS/MS), which is widely used in studying the metabolism of cancer and other diseases [16], was used based on its sensitivity, repeatability and high-throughput [17]. Moreover, the mathematical model of biomarkers, based on the alteration of multiple metabolites and analyzing the data by R programing, was reported to help diagnosis of breast cancer, and chronic graft-versus-host disease [18, 19]. It is feasible to establish the mathematical model of amino acid panel for AL diagnosis.

For establishing mathematical model of biomarkers, compared with R programing [18, 19], eXtreme Gradient Boosting (XGBoost), established by Chen, is proved to be higher accuracy and excellent generalization ability [20]. The number of the document of XGBoost folked on Github was more than 20,000. As it spreaded more and more, XGBoost was used to predict positive urinary tract infections and chemical-induced respiratory toxicity [21, 22].

Here, we used targeted LC–MS/MS to measure the amino acid profiles of PB between AL children and their matched control. The mathematical models were established and optimized using XGBoost algorithm. We then evaluated the models in another clinical cohort to assess their sensitivities, specificities and accuracies, to prove the advantageous performance of our model for distinguishing between children with AL and children with non-malignant hematologic diseases, who had similar clinical symptoms.

## Methods

### Enrolled patients and matched controls

**Fig. 1:**
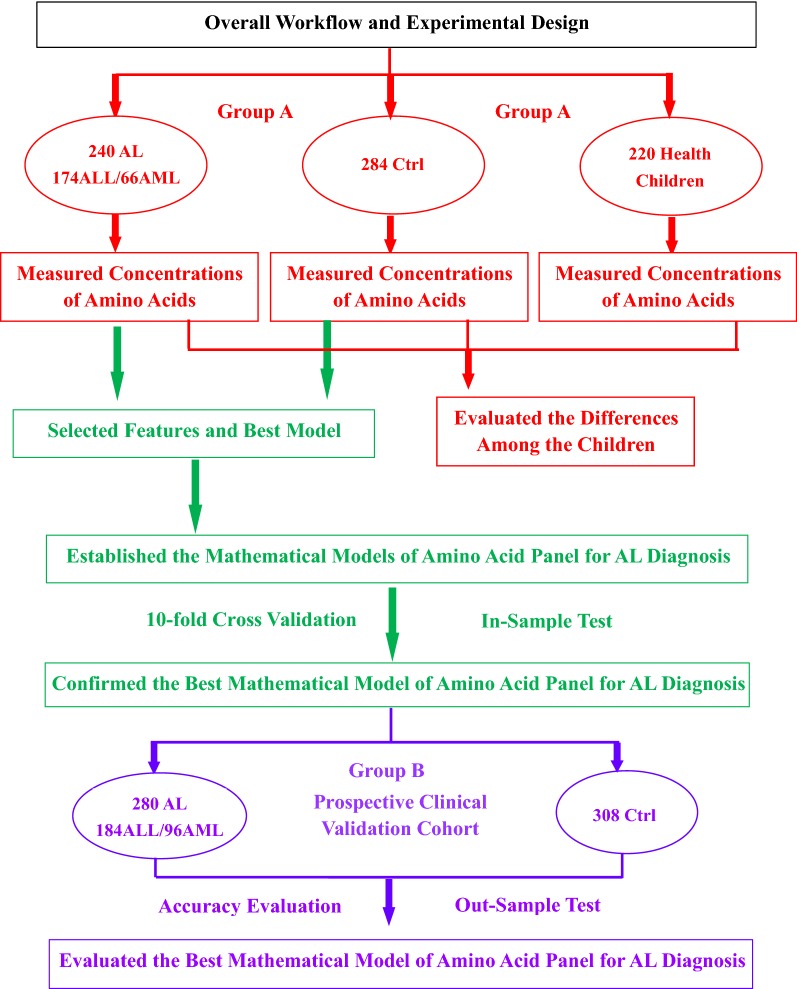
The overview of study design. In the phase of model establishment, 240 newly diagnosed AL children (ALL/AML = 174/66), 284 children with non-neoplastic hematological diseases and 220 healthy children were recruited for amino acids quantization with LC–MS/MS (red part). Based on the concentrations of 17 amino acids in all the patients and controls, we evaluated the differences among groups (red part) and the best model was established by Python-sklearn (green part). The model was then improved and verified by parameters adjusting and cross-validation (green part). Finally, another prospective independent cohort consisting of 280 newly diagnosed AL (ALL/AML = 184/96) and 308 children with non-neoplastic hematological diseases were used for further clinical verification as Out-Sample Test (purple part)

There were 520 newly diagnosed acute leukemia (AL) patients (ALL/AML = 358/162) recruited for this study and the inclusion criteria followed the AL diagnosis criteria in the 2016 edition of the World Health Organization (WHO) [23], and 592 children in their matched control group from April 2016 to March 2018. AL children, who were newly diagnosed and received normal diet (just avoiding high protein diet intake) 3 days before admission, were chosen in our study during the period. Children with missing clinical information related to MICM classification were not included in the study. The matched children controls were randomly chosen from patients with a non-malignant hematologic diseases, including anemia, infectious mononucleosis or thrombocytopenia, and received normal diet 3 days before admission in the same period and healthy children were chosen randomly from those who came to receive physical examination in the same period. Both matched healthy children (n = 220) and children with non-malignant hematologic diseases (n = 592) were used as controls to compare whether there was a difference among AL children, healthy children and children with non-malignant hematologic diseases. The sample size of controls were slightly larger than that of AL children (10–20% more) to ensure the data characteristics of control group were matched with that of leukemia group. The experimental design for this study was shown in Fig. 1. This project was approved by the institutional ethics board of the Children’s Hospital of Chongqing Medical University (CHCMU2015031). Informed consents were signed and obtained from the legal guardians of all patients.

### MICM stratification

Briefly, the French–American–British (FAB) classification standard for the morphological examination was used in this study [24]. For the immunological flow cytometry tests, BM cells from AL patients were incubated with specific antibodies (BD Biosciences, USA; Additional file 1: Table S1) and measured by Canto II flow cytometer (BD Biosciences, USA). The cytogenetic features of bone marrow cells were detected with Giemsa staining and karyotyping, and the tests for fusion genes were performed according to the manufacturer’s instructions (Yuanqi Bio-Pharm., Shanghai, China). The regime guidelines for AL patients were based on the 2016 edition of the World Health Organization (WHO) [23].

### Amino acid quantitation using targeted LC–MS/MS

Seventeen amino acids were quantified using LC–MS/MS (API 3200, Applied Biosystems) according to Turgeon’s report [25]. To ensure the quality of each dried blood spot, when the sample was collected, the standards and quality control were also spotted on filter papers at the same time. All the internal standards were prepared to achieve a series of gradient concentrations standards and spotted on filter paper (Whatman ProteinSaver 903). The standards, quality control and the sample was placed in a clean area of our laboratory for 2 h (1 h in summer) to dry, after that, it would be saved in a zip-lock bag at 4 °C until the experiment (no more than 3 h). The standards and quality control products were synchronized with the specimen. For experiment operations, briefly, metabolites from a dried blood spots were extracted with methanol. Internal standards (Cambridge Isotope Lab, USA) were added and samples were then dried under flowing nitrogen. The samples were butylated with HCl (50 µl) in each well. After evaporation under nitrogen, the samples were re-constituted in 100 µl of 80% acetonitrile. The samples (20 µl) were injected at 2-min intervals into a flowing stream of 80% acetonitrile. A neutral loss scan was used (m/z 102) for amino acids with a mass range of m/z 140–280. For the quality control of LC–MS/MS, all the internal standards and quality control products are kept with records to avoid overdue, and the internal standards and quality control products for each the amino acid were purchased from Cambridge Isotope Lab, and synchronizedly dealed with the specimen, to get the data for drawing the Levey-Jennings curve [26]. If the experiment was out of control, we perform it again. And if the deviation of the experiment was increased, it was adjusted according to quality control deviation. Because there were standard substance with isotope labelling for 17 amino acids and our targeted LC–MS/MS could only recognize isotope signals, we only detected 17 amino acids (shown in Table 1) in our study.

**Table 1:** Concentrations of amino acid among children in Group A

Amino acid	AL children n = 240	Ctrl n = 284	Healthy children n = 220	*p* value
Ala	134.59 ± 49.41	148.99 ± 47.90	144.87 ± 47.93	0.084
Asp	17.59 ± 8.58	14.17 ± 3.39	13.63 ± 2.77	< 0.001
Glu	27.94 ± 14.69	20.84 ± 4.95	29.75 ± 6.49	< 0.001
Met	18.35 ± 11.18	21.88 ± 10.17	16.24 ± 6.61	0.002
Phe	59.81 ± 23.23	35.96 ± 7.77	49.99 ± 28.98	< 0.001
Tyr	35.85 ± 14.88	31.17 ± 10.62	39.87 ± 18.55	0.001
Leu	55.81 ± 16.56	66.57 ± 15.64	62.82 ± 15.46	< 0.001
Trp	20.28 ± 13.15	14.77 ± 4.06	17.27 ± 5.18	< 0.001
Val	95.41 ± 28.42	103.12 ± 23.40	112.37 ± 29.90	0.001
Arg	60.68 ± 20.43	60.83 ± 15.51	66.12 ± 19.70	0.177
Cit	11.90 ± 4.44	16.45 ± 4.25	15.40 ± 5.93	< 0.001
Gly	80.60 ± 33.77	66.36 ± 14.91	69.04 ± 19.80	< 0.001
Orn	21.61 ± 4.88	24.23 ± 2.62	33.01 ± 3.08	< 0.001
Gln	16.45 ± 5.88	17.06 ± 2.80	6.47 ± 2.39	< 0.001
His	84.98 ± 79.31	73.50 ± 61.55	73.38 ± 22.13	0.357
Ser	9.99 ± 4.31	8.73 ± 1.67	11.10 ± 2.99	< 0.001
Thr	14.92 ± 7.72	14.52 ± 3.94	16.41 ± 6.75	0.215

Ala: alanine; Asp: aspartic acid; Glu: glutamic acid; Met: methionine; Phe: phenylalanine; Tyr: tyrosine; Leu: leucine; Trp: tryptophane; Val: valine; Arg: argnine; Cit: citrulline; Gly: glycine; Orn: ornithine; Gln: glutamine; His: histidine; Ser: serine; Thr: threonine

### Mathematical models establishment and feature selection

The mathematical models of the profile of 17 amino acids in dried serum dots from AL patients and matched controls, were established by support vector machine (SVM) [27], random forest (RF) [28] and XGBoost subsequently [20]. We only used the training set for the feature selection because it is critical for a model’s efficiency and performance. The concentrations of all the amino acids were normalized by zero-mean normalization. Considering the sample size we collected and avoiding overly complex model, any amino acid with Pearson correlation coefficient higher than 0.2 corresponding to the groups of children was chosen as a feature in the model. Simultaneously, if colinearity exhibit among different amino acids, we would choose only one amino acid, which had best Pearson correlation, as a feature.

### Model selection

To establish the best model, three classification algorithms (SVM, RF and XGBoost) were used and evaluated [20, 27, 28]. The classifiers were trained and evaluated by a tenfold cross-validation [27]. The final performance of each model was evaluated based on the averaging performance. The model would be chosen based on the comprehensive consideration of sensitivity, specificity, accuracy and volatility among cross-validation.

### Model development and validation

All clinical information and the altered concentration of amino acid panel determined by LC–MS/MS were analyzed using the Python-sklearn and SPSS. For models development, the patients of Group A (Fig. 1) were enrolled to establish models. The patients were randomly divided into training (80% samples) and validation (20% samples) sets. The models were trained using the training sets and subsequently used to predict a child with leukemia using the validation sets. The prediction accuracy was used to evaluate models by a tenfold cross-validation. To avoid over-fitting, learning_curve was introduced to evaluate whether algorithm was over-fitting at the statistical level firstly.

### Model assessment

The models were used to predict the patients of Group B (Fig. 1) to evaluate the models whether they were over-fitting depending on the accuracy of each model on Group B. There were 280 children with AL and 308 children with non-malignant hematologic disease included in the assessment. The stability of the final model, which was defined as “the ratio of the accuracy of Out-Sample Test to that of In-Sample Test”, was used to assess the performance of the final model.

### Analysis and statistics

The concentrations of amino acids in different groups were analyzed by one-way ANOVA. The efficacy of the models was further evaluated by McNemar’s test and ROC analysis. SPSS version 13.0 and Python version 3.6 were used, and the packages employed included “sklearn”, “seaborn”, “pandas”, “numpy” and “matplotlib”.

## Results

### Patients and clinical characteristics

The experimental design for this study and the characteristics of a total of 1332 children were enrolled in this study, including 520 newly diagnosed AL patients (ALL/AML = 358/162), 592 children in their matched control group and 220 healthy children, were also given (Fig. 1 and Additional file 1: Table S2). The initial 240 AL children and 284 children with a non-malignant hematologic diseases were assigned to Group A, and the 220 healthy children were also chosen in the same period. After model establishment, another 280 AL children and 308 children with a non-malignant hematologic diseases were chosen and assigned to Group B. There were no significant differences in the patients’ gender ratio and ages between the groups of AL and the matched control, nor WBC account and the percentage of blast cells in peripheral blood (BIPB) in the AL group. All related data were collected for each patient and control, and evaluated based on the same procedure.

### Feature selection and model selection

The concentrations of 17 amino acids in the serum from another 240 newly diagnosed AL patients (ALL/AML = 174/66), 284 matched control children and 220 healthy children were measured by targeted LC–MS/MS (Table 1). The levels of 13 amino acids (aspartic acid, glutamic acid, methionine, phenylalanine, tyrosine, leucine, tryptophane, valine, citrulline, glycine, ornithine, glutamine and serine) were statistically different among the AL children, controls and healthy children group, whereas other four amino acids (alanine, argnine, histidine and threonine), which didn’t show any statistical differences, were not enrolled in mathematical model.

**Fig. 2:**
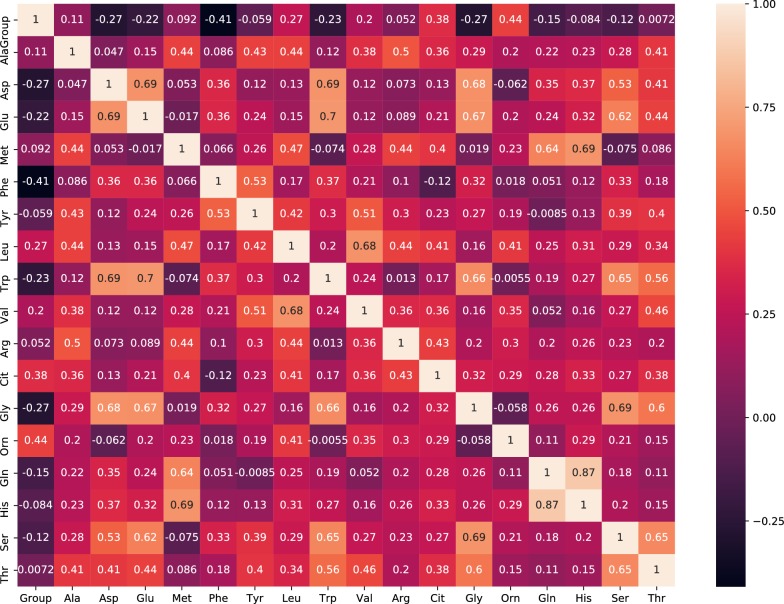
The heatmap of the Pearson correlation coefficients between each amino acid group. Ala: alanine; Asp: aspartic acid; Glu: glutamic acid; Met: methionine; Phe: phenylalanine; Tyr: tyrosine; Leu: leucine; Trp: tryptophane; Val: valine; Arg: argnine; Cit: citrulline; Gly: glycine; Orn: ornithine; Gln: glutamine; His: histidine; Ser: serine; Thr: threonine; Group: The classification of children (All the children were divided into three groups: AL children, controls and healthy children, so each child had a label. Because we would establish model under supervised learning protocol, we need to evaluate the correlation between every amino acid and each label. The value of each amino acid to Group was higher, which mean the correlation between the amino acid and the diagnosis of AL was closer.)

The eight amino acids (aspartic acid, glutamic acid, phenyl alanine, tryptophan, glycine, valine, citrulline and ornithine) were chosen to be included in the model for clinical diagnosis as each Pearson correlation coefficient was higher than 0.2 (Fig. 2) and each was related with cell proliferation, apoptosis or drug treatment of different cancers [9–15].

The data of the eight amino acids were used to develop models based on the three classification algorithms (SVM, RF and XGBoost). Accuracy, sensitivity, specificity and area under the curve (AUC) of the three algorithms were shown in Table 2. Although XGBoost had the best sensitivity, accuracy and AUC, and its specificity was also better than RF, but each indicator of XGBoost was not better than SVM and RF. All the three algorithms should be optimized and evaluated further.

**Table 2:** The performance of models on AL diagnosis for In-Sample Test

	SVM	RF	XGBoost
Sensitivity (%)	92.23 ± 4.32	94.44 ± 5.27	95.86 ± 4.21
Specificity (%)	94.43 ± 3.77	91.76 ± 4.85	94.21 ± 4.96
Accuracy (%)	87.24 ± 4.23	88.76 ± 5.11	90.23 ± 4.89
AUC^a^	0.812 ± 0.036	0.821 ± 0.032	0.828 ± 0.035

SVM: support vector machine; RF: random forest; XGboost: eXtreme Gradient Boosting; AUC: area under curve

^a^ROC analysis

### Parameter optimization in models for AL diagnosis and validation

To establish a better model for AL diagnosis, we focused on optimizing several key parameters. For SVM, the parameters included C, kernel, degree, gamma, coef0, max_iter; For RF, the parameters included n_estimators, max_depth, min_samples_split, min_samples_leaf, max_leaf_nodes; For XGBoost, the parameters included learnin_rate, n_estimators, max_depth, gamma, subsample, colsample_bytree and nthread. The optimized parameters were confirmed by performing tenfold cross validation on the training and validation data sets [27]. The final models were also verified with ROC and AUC by cross-validation (Table 3). The mean AUC was 0.848 (95% CI 0.819 to 0.877) for SVM. The mean AUC was 0.834 (95% CI 0.811 to 0.857) for RF. The mean AUC was 0.856 (95% CI 0.809 to 0.923) for XGBoost.

**Table 3:** The cross-validation of best model for each algorithm on AL diagnosis for In-Sample Test

	SVM	RF	XGBoost
Mean of accuracy (%) (95% CI)	89.84 (84.72, 94.96)	90.12 (84.67, 95.57)	91.35 (87.05, 95.65)
Mean of AUC (95% CI)	0.848 (0.819, 0.877)	0.834 (0.811, 0.857)	0.856 (0.809, 0.923)

### Evaluation of amino acid panels for AL diagnosis

**Fig. 3:**
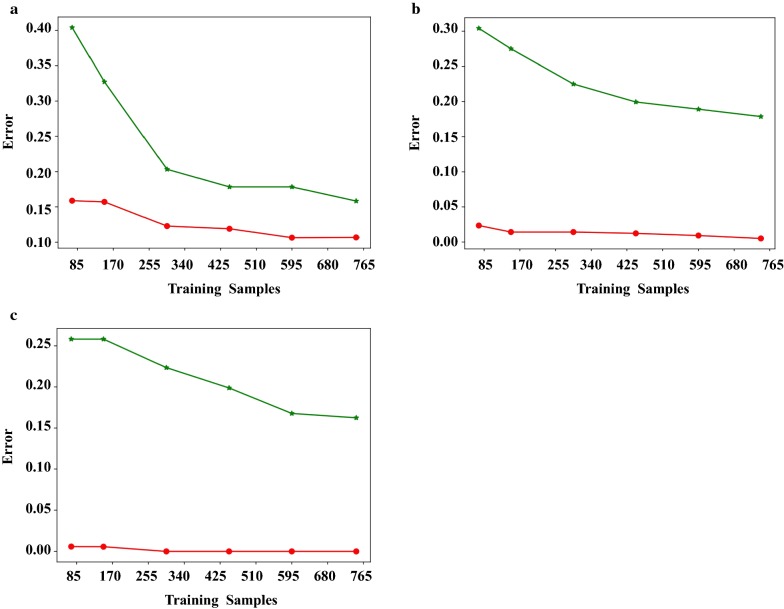
The learning-curve for the three algorithm. **a** The learning-curve for SVM; **b** the learning-curve for RF; **c** the learning-curve for XGBoost; red curve stood for training set and green curve stood for testing set

Before assess the accuracy of the models, all of them should be proved whether they were over-fitting by learning_curve (Fig. 3). It was obvious that the difference of errors between the testing samples and training samples in each model converged as the number of samples increased, which mean all the models we built were not over-fitting at the statistical level.

To further assess the accuracy of the models, they were evaluated according to the reported protocol [29]. We further validated the models on Group B. There were 280 newly diagnosed AL patients (ALL/AML = 184/96) and 308 children in their matched control group, who were included in Group B (Table 1). There was no significant difference between the conventional methods and each model on AL diagnosis according to Table 4 (*p* > 0.05). The sensitivity, specificity, accuracy and AUC of the models were shown in Table 4. The sensitivity of SVM, RF and XGBoost for Out-Sample Test was 84.64%, 82.50% and 90.00% respectively. The specificity of SVM, RF and XGBoost for Out-Sample Test was 85.06%, 87.66% and 88.96% respectively. The accuracy of SVM, RF and XGBoost for Out-Sample Test was 84.86%, 85.20% and 89.46% respectively. The AUC of SVM, RF and XGBoost for Out-Sample Test were 0.797, 0.803 and 0.830 respectively. Comparing with the accuracies of these models for In-Sample Test (Table 3), the accuracies of SVM, RF and XGBoost for Out-Sample Test were all in 95% confidence interval. It was another evidence to prove that all of our models were not over-fitting. The sensitivity, specificity and accuracy of XGBoost were the best among the three models (Table 4). The generalization ability of each model, which was defined as “the accuracy of Out-Sample Test/the mean accuracy of In-Sample Test” in our study, was 0.945 (84.86%/89.84%), 0.945 (85.20%/90.12%), 0.979 (89.46%/91.35%) respectively. XGBoost model also had the best generalization ability.

**Table 4:** The validation of models on AL diagnosis for Out-Sample Test

	Diagnosis (model/clinical diagnosis)				χ^2^	*Kappa* value	*p* value	AUC^i^
	+/+ ^a^	∓^b^	±^c^	−/−^d^				
Result-SVM^h^	237	43	46	262	0.1011	0.697	0.751	0.788
Sensitivity^e^ (%)	84.64							
Specificity^f^ (%)	85.06							
Accuracy^g^ (%)	84.86							
Result-RF^h^	231	49	38	270	1.3908	0.703	0.238	0.803
Sensitivity^e^ (%)	82.50							
Specificity^f^ (%)	87.66							
Accuracy^g^ (%)	85.20							
Result-XGB^h^	252	28	34	274	0.2903	0.789	0.446	0.830
Sensitivity^e^ (%)	90.00							
Specificity^f^ (%)	88.96							
Accuracy^g^ (%)	89.46							

SVM: support vector machine; RF: random forest; XGB: XGBoot; FN: false negative; FP: false positive; AUC: area under curve

^a^Our model or clinical diagnosis were both positive-children were with leukemia

^b^Our model diagnosed children as normal, but the clinical diagnosis of them was leukemia

^c^Our model diagnosed children as leukemia, but the clinical diagnosis of them was normal

^d^Our model or clinical diagnosis were both negative, and children were normal

^e^Number of +/+ for each model/(number of +/+ for each model plus number of ∓ for each model) × 100%

^f^Number of −/− for each model/(number of −/− for each model plus number of ± for each model) × 100%

^g^(Number of −/− for each model plus number of +/+ for each model)/588 × 100%

^h^McNemar’s test

^i^ROC analysis

Next, we compared the true positive and negative prediction performance on XGBoost model with morphological tests (Table 5). The performance of XGBoost was much better than that of morphological tests alone. Furthermore, if we combine morphological tests and XGBoost model to diagnose AL in clinical application, it would greatly reduce the false negative ratio of morphological tests and improve the diagnosis efficacy of XGBoost model.

**Table 5:** The true positive and negative prediction performance of morphology and XGBoost model in Group B

	Diagnosis (model/clinical diagnosis)				*Kappa* value	*p* value	AUC^e^
	+/+^a^	∓^b^	±^c^	−/−^d^			
M	268	12	86	222	0.670	< 0.001	0.742
X	252	28	34	274	0.789	0.720	0.830
M + X	262	18	26	282	0.850	0.523	0.872

McNemar’s test

M: morphology; X: XGBoost model; AUC: area under curve

^a^Our model or clinical diagnosis were both positive-children were with leukemia

^b^Our model diagnosed children as normal, but the clinical diagnosis of them was leukemia

^c^Our model diagnosed children as leukemia, but the clinical diagnosis of them was normal

^d^Our model or clinical diagnosis were both negative, and children were normal

^e^ROC analysis

## Discussion

The classical diagnosis of AL is usually based on the MICM information of patients’ bone marrow [3] and the relationship between amino acid profile and AL diagnosis has not been established previously. Here, we developed new strategies to diagnose AL by measuring concentrations of PB amino acids with LC–MS/MS and further data mining. Additionally, all the models for AL diagnosis were verified by tenfold cross validation and used to assist AL diagnosis.

As others’ report, SVM maps the input data into a high-dimensional feature space through some kernel functions and constructs an optimal separating hyperplane in this space [22], but it could require more computation time; RF is considered to be more accurate and robust than decision trees and the most important advantages of it is that it can handle a large number of features without overfitting, and can give an estimate of the importance of the features [22]; XGBoost is a new implementation of the gradient tree boosting technique and has been tested in a series of datasets, achieving high accuracy and requiring much less computation time than deep neural nets [22], so we chose these three algorithms as candidates. Because XGBoost algorithm used the second order Taylor expansion [20], it could get a more accurate result on predicting than normal gradient tree boosting algorithm and it has a better convergence effect than SVM and RF. In our study, all the three models were not overfitting and the generalization ability of each of them (more than 94% samples would be correctly predicted) deserved further clinical application. According to our data, there was no significant differences on accuracy and AUC among the three models after parameter optimization during training process, but the sensitivity, specificity and accuracy of XGBoost were better than SVM and RF (Table 4). XGBoost had the best generalization ability among them, which is the most important character of model, in the Out-Sample Test. Above all, we recommend XGBoost to be the auxiliary diagnostic model at present. Combining the three models but not limited to them to establish artificial neural network for the diagnosis of AL would be our next step.

According to Table 4, the sensitivity and specificity of XGBoost were more than 88.96% comparing with traditional protocol on AL diagnosis and there was no statistic significant difference between them (*p *> 0.05). Simultaneously, the new model we established does not aim to replace the conventional methods. The most important contribution of the strategy is that it could help doctors distinguish acute leukemia patients from others hematological diseases which may appear similar phenotype as leukemia in an easier way and faster, so that they can determine treatment plan in time, not waiting for days to make a decision. It would be helpful for doctors from the department of hematology to screen suspicious patients, especially for outpatient. Considering the accuracy of our model (88.96%), it is good enough to help doctors from the department of hematology as an auxiliary diagnostic method.

There were three advantages of our new model comparing with conventional assays. Firstly, for the time-consuming of assays, the conventional laboratory assays to diagnose AL including morphological tests, karyotype, flow cytometry and molecular detections [2]. It usually needs at least 3 days to diagnose AL. Our new strategy based on LC–MS/MS and mathematical model, which only took 4–6 h to complete analysis; Secondly, for the expense, different kinds of antibodies and professional assay kits were needed for flow cytometry and molecular detections (The prices for antibodies and kits could refer to BD Biosciences and Yuanqi Bio-Pharm), it took approximate $250 for each child to complete the assays in China, however, the main expense of our new strategy is approximate $20 for each child in China; At last, for sample collection and operation, bone marrow should be collected to perform karyotype, flow cytometry and molecular detections for conventional laboratory assays, and karyotype would consume a lot of manual operation, but only PB sample should be collected for our model, which is much easier to collect and less painful, especially for children [3], and the main assay in our model, LC–MS/MS, is a automation technique requiring little manual operation. Based on the statement above, our strategy is faster, cheaper and more convenient way than conventional strategy (Table 6). As the combination results shown in Table 5, combining XGBoost model and morphological tests would gain a better predictive power. It was another evidence to prove that our model was absolutely related to AL, only the exact mechanism between the amino acid profile and AL had not been clarified.

**Table 6:** The comparison between new strategy and conventional methods

	New strategy	Conventional methods
Time-consuming	4–6 h	3 days
Expense	$20 per child	$250 per child
Sample collection	Peripheral blood (easy to collect)	Bone marrow (hard to collect)

We also tried to establish models to predict the prognosis of AL patients, but the result was unsatisfied with the following reasons. Firstly, the prognosis of AL patients was not only determined by risk classification, but also influenced by the status of compliance of medical treatment. Our model could not take the therapeutic status into account. Secondly, the prognosis of AL has improved to a long-term survival rate of 89% [30]. Our results showed no significant difference because there were few ALL patients die during our observation stages.

We also attempted to establish a mathematical model of amino acid profile to separate ALL and AML. However, the model was not able to evaluate its actual performance. There were two main reasons that our model could not distinguish ALL and AML. Firstly, AML samples were dispersed because of the high heterogeneity of AML [31], resulting in few samples (< 25) in each subtype of AML (Additional file 1: Table S2); Secondly, there was a high abandon rate among AML patients with less clinical information. Based on the above reasons, the sample size of AML was not enough for establishing model. Moreover, we tried to investigate if there was a difference on amino acid concentration among various karyotyping or fusion gene groups in ALL, but there was no significant difference among them (Additional file 1: Tables S3 and S4). There was no significant difference among them, so we did not build model to analyze it through SVM, RF or XGBoost algorithm.

The new biomarkers using small molecule metabolites for diagnosis is a hot area for different cancers. For example, a biomarker panel including phenylacetic acid, l-fucose, caprylic acid, acetic acid, propionic acid and glycine achieved good performance with the sensitivity of 80% and specificity of 100% for predicting small cell lung cancer [32]. A diagnosis panel containing circulating tumor cell number and lactate dehydrogenase level was found to be a surrogate for survival at the individual-patient level in metastatic castration-resistant prostate cancer [33]. A series of metabolites, including d-mannose, palmitic acid, stearic acid, etc., which are present in the disease state, were identified as candidate biomarkers for B-ALL diagnosis, but no prediction model was used [34]. To our best knowledge, there was no report focused on amino acid panel for the diagnosis of leukemia. Our study is the first attempt to establish a model to link amino acids profile and children acute leukemia.

This study mainly focused on the amino acids profile to establish the mathematical models for AL diagnosis. However, the underlying mechanism of amino acid metabolism in AL needs further investigation. According to the WHO guidelines for diagnosis and genotype of leukemia (2016 edition) [23] and previous reports [35], the molecular variation of patients is very important for predicting the prognosis of AL. It is necessary to get more information of AL patients by next-generation sequence, including whole genome sequencing, transcriptome sequencing, and RNA sequencing [36, 37], to create new cross-omics models, which integrate genomics and metabolomics to provide all the information of enzymes in the pathways related to leukemia.

In addition, combining metabolomics approach and data mining to establish prediction models has been demonstrated as a strategy potentially useful for diagnosis or prognosis in different diseases [38, 39]. Although we demonstrated the precise diagnosis of leukemia in this study using the same approach, the model will be more accurate and reliable if a larger sample size is used, especially multi-center study, to refine the models in the future.

## Conclusions

In summary, based on the PB amino acids profile, we developed a mathematical model to diagnose children AL. There was no significant difference on the effect of children AL diagnosis between our new model and the traditional protocol. Simultaneously, the model is a faster, cheaper and more convenient way than conventional methods. It could benefit the clinical practice for children AL diagnosis and treatment.

## Additional file

**Additional file 1: Table S1.** The Surface Markers Detected by Flow Cytometry. **Table S2.** The characteristics of all patients in the sections. **Table S3.** Concentrations of amino acid among ALL children in different risk level based on chromosomal detection. **Table S4.** Concentrations of amino acid among ALL children in different risk level based on fusion gene detection.

## Abbreviations

AL: acute leukemia
ALL: acute lymphoblastic leukemia
AML: acute myelocytic leukemia
MICM: morphological, immunological, cytogenetic and molecular
BIPB: blast cells in peripheral blood
WBC: white blood cell
PB: peripheral blood
ROC: receiver operating characteristic
LC–MS/MS: liquid chromatography mass spectrometry
DBS: dried blood spot
WHO: World Health Organization
FAB: French–American–British
CCLG: China Children’s Leukemia Group
XGBoost: eXtreme Gradient Boosting
SVM: support vector machine
RF: random forest
QC: quality control
AUC: area under curve
