# Supplementary material for: Mathematical models of amino acid panel for assisting diagnosis of children acute leukemia
Source: J Transl Med. 2019 Jan 23;17:38. doi: 10.1186/s12967-019-1783-9 (PMC6343345; doi:10.1186/s12967-019-1783-9)
Supplement: Supplementary file 1 — Additional file 1: Table S1. The Surface Markers Detected by Flow Cytometry. Table S2. The characteristics of all patients in the sections. Table S3. Concentrations of amino acid among ALL children in different risk level based on chromosomal detection. Table S4. Concentrations of amino acid among ALL children in different risk level based on fusion gene detection. [file 12967_2019_1783_MOESM1_ESM.docx]

**Table S1. The Surface Markers Detected by Flow Cytometry**

| **ALL Surface Markers** | | **AML Surface Markers** | | **Other Surface Marker** |
| --- | --- | --- | --- | --- |
| CD10 | CD2 | CD13 | cMPO | CD34 |
| CD19 | CD3 | CD14 | cCD41a | HLA-DR |
| CD20 | cCD3 | CD15 | cCD61 | CD56 |
| cCD79a | CD4 | CD16 |  | CD38 |
| cIgM | CD5 | CD33 |  | CD36 |
| cCD22 | CD7 | CD64 |  |  |
| nuTDT | CD8 | CD117 |  |  |

**Table S2. The characteristics of all patients in the sections**

|  | **Group A** | | **Group B** | |  |
| --- | --- | --- | --- | --- | --- |
|  | **AL** (n=240)  ALL/AML  (174/66) | **Ctrl**  (n=284) | **AL** (n=280)  ALL/AML  (184/96) | **Ctrl**  (n=308) | **Healthy Children**  (n=220) |
| **Gender** |  |  |  |  |  |
| Male | 102/42 | 170 | 110/58 | 184 | 130 |
| Female | 72/24 | 114 | 74/38 | 124 | 90 |
| **Age (years)** |  |  |  |  |  |
| 1<Age<10 | 130/46 | 188 | 138/48 | 218 | 156 |
| Age≤1 or Age≥10 | 44/20 | 96 | 46/48 | 90 | 64 |
| **WBC (×10^9/L)** |  |  |  |  |  |
| <50 | 154/36 | 284 | 162/48 | 308 | 220 |
| ≥50 | 20/30 | NA | 22/48 | NA | NA |
| **BIPB (%)** | 49.38±28.69/  48.26±23.77 |  | 48.55±19.32/  47.38±19.69 |  |  |
| **Immunological Features** |  |  |  |  |  |
| Mature B/M2 | 6/22 |  | 14/38 |  |  |
| Pre B/M3 | 18/8 |  | 32/22 |  |  |
| Pro B/M4 | 24/8 |  | 22/10 |  |  |
| Common B/M5 | 130/10 |  | 116/18 |  |  |
| M6 | 14 |  | 8 |  |  |
| **Cytogenetic Features ^b^** | **n=150** |  |  |  |  |
| Group I | 54/4 |  | 56/10 |  |  |
| Group II | 16/4 |  | 26/14 |  |  |
| Group III | 60/12 |  | 102/72 |  |  |
| **Molecular Features ^c^** | **n=150** |  |  |  |  |
| Group I | 44/4 |  | 48/12 |  |  |
| Group II | 24/4 |  | 40/14 |  |  |
| Group III | 62/12 |  | 96/70 |  |  |
| **Prednisone Sensitivity (D8) ^d^** | **n=142** |  |  |  |  |
| Sensitive | 98/6 |  | 128/66 |  |  |
| Insensitive | 24/14 |  | 56/30 |  |  |
| **MRD (D19) ^e^** | **n=122** |  |  |  |  |
| <1% | 98/8 |  | 154/38 |  |  |
| ≥1% | 10/6 |  | 30/58 |  |  |
| **Extramedullary Infiltration** | 4/0 |  | 0/0 |  |  |

**Note:**

**a:** Children with other hematologic diseases, such as anemia, infectious mononucleosis and thrombocytopenia.

**b**: The standard of cytogenetic classification: Group I: trisomy 21, hypodiploid (the number of chromosomes <44), there is a t(1:19), t(10:11) or t(9:22) rearrangement; Group II: hyperdiploid (the number of chromosomes ≥50); Group III: normal karyotype, inversion of chromosome 16.

**c**: The standard of molecular features classification: Group I: BCR/ABL, CBFβ/MYH11, MLL/AF4, MLL/AF9, E2A/PBX1, EVI1 positive, PML/RARa, FLT3-ITD; Group II: AML1-ETO, TEL/AML1; Group III: without fusion genes.

**d:** Whether the treatment of prednisone was sensitive or not on the 8^th^ day.

**e:** Whether the MRD was above 1% or not on the 19^th^ day.

Abbreviations: WBC, white blood cell; BIPB, blast cells in peripheral blood; MRD, minimal residual disease.

**Table S3. Concentrations of amino acid among ALL children in different risk level based on chromosomal detection**

| **Amino Acid** | **Group I**  **n=35** | **Group II**  **n=13** | **Group III**  **n=126** | ***p* value** |
| --- | --- | --- | --- | --- |
| Ala | 133.41±50.41 | 142.86±54.66 | 124.99±57.83 | 0.883 |
| Asp | 17.09±7.79 | 18.34±8.63 | 19.48±11.14 | 0.669 |
| Glu | 27.07±13.68 | 27.75±14.48 | 27.06±14.47 | 0.301 |
| Met | 18.38±11.31 | 19.73±12.31 | 19.81±13.32 | 0.740 |
| Phe | 59.09±23.29 | 63.01±23.23 | 56.24±25.54 | 0.698 |
| Tyr | 35.78±14.99 | 36.47±16.45 | 38.08±16.38 | 0.393 |
| Leu | 55.37±16.22 | 55.63±15.54 | 56.23±16.43 | 0.586 |
| Trp | 19.57±11.77 | 19.92±12.99 | 26.64±15.27 | 0.365 |
| Val | 94.71±28.71 | 95.37±25.81 | 92.26±30.11 | 0.477 |
| Arg | 60.46±21.04 | 65.06±18.98 | 57.00±23.35 | 0.433 |
| Cit | 11.831±4.29 | 12.56±4.46 | 13.42±6.54 | 0.379 |
| Gly | 78.24±31.19 | 82.52±34.52 | 76.63±35.26 | 0.927 |
| Orn | 21.76±5.53 | 21.72±4.55 | 27.63±11.26 | 0.237 |
| Gln | 16.53±7.21 | 17.25±7.13 | 21.50±13.00 | 0.677 |
| His | 81.74±78.38 | 89.73±88.17 | 78.98±78.67 | 0.410 |
| Ser | 9.64±3.75 | 10.07±4.25 | 10.55±4.70 | 0.675 |
| Thr | 14.55±6.75 | 15.11±8.23 | 14.99±7.70 | 0.667 |

**Abbreviations:** Ala, Alanine; Asp, Aspartic acid; Glu, Glutamic acid; Met, Methionine; Phe, Phenylalanine; Tyr, Tyrosine; Leu, Leucine; Trp, Tryptophane; Val, Valine; Arg, Arginine; Cit, Citrulline; Gly, Glycine; Orn, Ornithine; Gln, Glutamine; His, Histidine; Ser, Serine; Thr, Threonine.

The standard of cytogenetic classification: Group I: trisomy 21, hypodiploid (the number of chromosomes <44), there is a t(1:19), t(10:11) or t(9:22) rearrangement; Group II: hyperdiploid (the number of chromosomes ≥50); Group III: normal karyotype, inversion of chromosome 16.

**Table S4. Concentrations of amino acid among ALL children in different risk level based on fusion gene detection**

| **Amino Acid** | **Group I**  **n=31** | **Group II**  **n=21** | **Group III**  **n=122** | ***p* value** |
| --- | --- | --- | --- | --- |
| Ala | 134.37±50.53 | 134.82±47.55 | 124.99±57.83 | 0.328 |
| Asp | 17.23±7.94 | 15.86±5.84 | 19.48±11.14 | 0.664 |
| Glu | 27.32±13.98 | 25.25±10.85 | 27.06±14.47 | 0.065 |
| Met | 18.46±11.24 | 18.44±11.45 | 19.81±13.32 | 0.822 |
| Phe | 59.06±23.31 | 58.49±22.37 | 56.24±25.54 | 0.429 |
| Tyr | 35.88±14.92 | 35.74±14.83 | 38.08±16.38 | 0.426 |
| Leu | 55.69±16.62 | 56.16±16.27 | 56.23±16.43 | 0.443 |
| Trp | 20.11±13.09 | 17.70±8.69 | 26.64±15.27 | 0.280 |
| Val | 94.86±29.13 | 96.27±28.92 | 92.26±30.11 | 0.190 |
| Arg | 60.15±20.93 | 62.18±20.79 | 57.00±23.35 | 0.817 |
| Cit | 11.90±4.40 | 11.53±4.14 | 13.42±6.54 | 0.119 |
| Gly | 78.89±32.11 | 74.74±25.92 | 76.63±35.27 | 0.404 |
| Orn | 21.79±5.55 | 21.53±5.04 | 27.63±11.27 | 0.063 |
| Gln | 16.67±7.23 | 14.03±5.14 | 21.50±13.00 | 0.741 |
| His | 83.06±79.73 | 73.79±76.12 | 78.98±78.67 | 0.781 |
| Ser | 9.79±4.01 | 9.18±3.15 | 10.55±4.70 | 0.750 |
| Thr | 14.89±7.68 | 13.84±5.92 | 15.00±7.70 | 0.247 |

**Abbreviations:** Ala, Alanine; Asp, Aspartic acid; Glu, Glutamic acid; Met, Methionine; Phe, Phenylalanine; Tyr, Tyrosine; Leu, Leucine; Trp, Tryptophane; Val, Valine; Arg, Arginine; Cit, Citrulline; Gly, Glycine; Orn, Ornithine; Gln, Glutamine; His, Histidine; Ser, Serine; Thr, Threonine.

The standard of molecular features classification: Group I: BCR/ABL, CBFβ/MYH11, MLL/AF4, MLL/AF9, E2A/PBX1, EVI1 positive, PML/RARa, FLT3-ITD; Group II: AML1-ETO, TEL/AML1; Group III: without fusion genes.
